# Commissioning cranial single‐isocenter multi‐target radiosurgery for the Versa HD

**DOI:** 10.1002/acm2.13223

**Published:** 2021-03-23

**Authors:** Cory Knill, Raminder Sandhu, Robert Halford, Michael Snyder, Zachary Seymour

**Affiliations:** ^1^ Department of Radiation Oncology Beaumont Health Royal Oak MI 48073 USA

**Keywords:** Elements Multiple Brain Mets SRS, Versa HD, SRS

## Abstract

**Purpose:**

Brainlab’s Elements Multiple Brain Mets SRS (MBMS) is a dedicated treatment planning system for single‐isocenter multi‐target (SIMT) cranial stereotactic radiosurgery (SRS) treatments. The purpose of this study is to present the commissioning experience of MBMS on an Elekta Versa HD.

**Methods:**

MBMS was commissioned for 6 X, 6 FFF, and 10 FFF. Beam data collected included: output factors, percent depth doses (PDDs), diagonal profiles, collimator transmission, and penumbra. Beam data were processed by Brainlab and resulting parameters were entered into the planning system to generate the beam model. Beam model accuracy was verified for simple fields. MBMS plans were created on previously treated cranial SRS patient data sets. Plans were evaluated using Paddick inverse conformity (ICI), gradient indices (GI), and cumulative volume of brain receiving 12 Gy. Dosimetric accuracy of the MBMS plans was verified using microDiamond, Gafchromic film, and SRS Mapcheck measurements of absolute dose and dose profiles for individual targets. Finally, an end‐to‐end (E2E) test was performed with a MR‐CT compatible phantom to validate the accuracy of the simulation‐to‐delivery process.

**Results:**

For square fields, calculated scatter factors were within 1.0% of measured, PDDs were within 0.5% past dmax, and diagonal profiles were within 0.5% for clinically relevant off‐axis distances (<10 cm). MBMS produced plans with ICIs < 1.5 and GIs < 5.0 for targets > 10 mm. Average point doses of the MBMS plans, measured by microDiamond, were within 0.31% of calculated (max 2.84%). Average per‐field planar pass rates were 98.0% (95.5% minimum) using a 2%/1 mm/10% threshold relative gamma analysis. E2E point dose measurements were within 1.5% of calculated and Gafchromic film pass rates were 99.6% using a 5%/1 mm/10% threshold gamma analysis.

**Conclusion:**

The experience presented can be used to aid the commissioning of the Versa HD in the Brainlab MBMS treatment planning system, to produce safe and accurate SIMT cranial SRS treatments.

## 1 Introduction

Elements Multiple Brain Mets SRS (MBMS) is a site‐specific planning system for treating multiple cranial targets that was developed by Brainlab (Brainlab, Munich, Germany). Unlike conventional planning systems that are designed to treat a wide range of anatomical sites, MBMS creates single‐isocenter multi‐target (SIMT) linac based cranial stereotactic radiosurgery (SRS) plans, using non‐coplanar dynamic conformal arcs. SIMT has the potential to create plans with similar organ at risk (OAR) sparing and target coverage, while reducing treatment times compared with multiple single‐target plans.^1^, ^2^, ^3^, ^4^ The specificity of the MBMS allows for an optimization algorithm that can focus on important cranial SRS metrics. The optimizer can also overcome typical linac based cranial SRS planning shortfalls, like the bridging of dose between two targets.

One drawback of the specificity is that the commissioning physicist is unable to perform an AAPM Task Group 119 type test of the system to compare their commissioning results for various anatomical sites to other institutions.^5^ Furthermore, MBMS may be commissioned at the start of an institution’s linac‐based cranial SRS implementation so there may not be any internal data for comparison. In this work, the MBMS commissioning experience on an Elekta Versa HD (Elekta AB, Stockholm, Sweden) will be presented which can be used for guidance as well as a baseline for comparison.

## 2 Methods

### 2.1 Generating beam model

Beam model measurements included: PDDs, profiles, scatter factors, collimator transmission, and dynamic leaf shift. Data collection followed Task Group 106 methodology.^6^ All measurements were made in Sun Nuclear’s (Sun Nuclear Corporation, Melbourne, FL) 3D water tank with Sun Nuclear’s 0.125 cc chamber, EDGE detector, or PTW’s (PTW‐Freiburg, Freiburg, Germany) microDiamond detector. A SNC 0.125 cc chamber was used as a reference chamber to normalize the data for fluctuations in linac output when scanning profiles and PDDs.

The water tank was setup to the central axis of the beam using a ray tracing procedure. For profile measurements, the tank was shifted 0.25 cm (1/2 leaf width) in the jaw direction, so the detectors intersected a multi‐leaf collimator (MLC) leaf tip instead of junction between two MLC leaves. For output measurements, a Daisy‐Chain method was used to calibrate the microDiamond for small field measurements.^7^ The SNC 0.125 cc was used to measure output factors down to a 3.0 cm field size, after which it was cross calibrated to the microDiamond chamber to measure output factors down to 1.0 cm. microDiamond measurement were performed without corrections, which is examined in the discussion.

Beam data were collected for energies 6 X, 6 FFF, and 10 FFF. The measured data were processed by Brainlab to calculate leaf shifts, tongue and groove sizes, source functions, and radial factors.

After beam model parameters were measured and calculated, machine models were created for each energy. Machine models require department specific parameters (machine name, coordinate convention, etc.) along with machine specific information such as dose rate and maximum gantry speed. The machine type parameters were collated from three sources: a) Versa HD manuals, b) Monaco manuals (provided by Elekta), and c) settings in existing hospital planning systems (Pinnacle). Following the creation of the machine model, the energy specific beam models were created for the three energies. Prior to final saving of the model, the system performed a secondary check for the data to help protect against non‐realistic values.

### 2.2 Validation

AAPM Task Group 53 was used to guide the treatment planning system (TPS) validation.^8^ The data transfer from Elements to the record and verify system (Mosaiq) was tested using various test plans. Subsequent data transfer to the linac and on‐board imagers was tested. Data fidelity was checked at each step of the transfer.

Initial validation of the MBMS version 1.5 beam model was done using the Beam Model Verification module, included in the Elements software, which allows the calculation of single fields on phantoms. Dose was calculated with a Pencil Beam Algorithm utilizing a 1 mm grid size. A virtual water phantom with density 1.0 g/cm^3^, simulating a water tank, was generated in Matlab and imported into Elements. The point dose, output factors, depth dose, and profiles were calculated using the same geometry as the commissioning measurements and verified against measured data.

Following beam model verification, the validation of typical clinical deliveries was performed. Since Elements is a template‐based software, various prescription and beam arrangement template protocols were generated to cover the range of expected clinical cases. Prescription protocols of 19 Gy × 1 fx, 8 Gy × 3 fx, and 6 Gy × 5 fx were created with various minimum target coverages of 95%, 97%, and 99% for a total of nine protocols. Beam templates for 2, 3, and 5 different couch angles were created. Two version of each protocols were created: a) all the couch angles on one side of the gantry, b) couch angles on both sides with symmetrical arrangement. MBMS automatically mirrors one‐sided protocols if the target is on the other side of the brain, therefore the generation of both left and right sided protocols was not needed.

To test the protocols, previously treated cranial SRS patients treated within the hospital system, were re‐planned in MBMS. All initial plans were 1 fraction treatments with the same prescriptions ranging from 15 Gy to 20 Gy covering 95% of the target. Fourteen clinical targets were studied ranging from 0.27 cc to 7.32 cc. Plans had between 2‐5 couch angles and 1‐5 targets. Each plan was recalculated for three energies: 6 X, 6 FFF, and 10 FFF. While it was known that 10FFF would not be used for cranial treatments, the energy was commissioned in anticipation of different anatomical Elements (ex. Spine). Regardless, it is recommended that at least two energies be commissioned simultaneously to allow cross‐comparison between results, which can be helpful with troubleshooting any inconsistencies that arise during validation.

Plan quality was evaluated using Paddick inverse conformity index (ICI), gradient index (GI), and Brain V_12 Gy_ (volume of normal brain getting dose of 12 Gy or more) to get an understanding of the limits of the system.^9^, ^10^ A subset of plans (volumes ranging from 0.89 cc to 7.32 cc) were exported to Mosaiq for dosimetric validation on the Versa HD. Dose validation was performed using a combination of Gafchromic film, microDiamond point dose measurements, and SRSMapcheck. All measurements were done using StereoPHAN with proper inserts. To setup the StereoPHAN in MBMS, multiple CTs were acquired will the different measurement inserts using a Philips Brilliance Big Bore CT scanner (Andover, MA) with 1mm slice thickness. The StereoPHAN was indexed to the CT couch while scanning with different inserts to prevent movement. This allowed for easy comparison of cross‐modality measurements (ex. Gafchromic Film and SRSMapcheck) using the same DICOM coordinates. The phantom was imported into MBMS and assigned a uniform density of 1.20 g/cm^3^ as specified by the StereoPHAN manual.

Initial dose validation of the phantom was performed by measuring simple square fields on the Versa HD at various gantry/couch angles. This confirmed the model within the TPS was correct and also verified the angular and field size dependencies of the various detectors matched manufacture’s specifications.

Subsequent dose validation was performed for MBMS plans. When measuring single target plans, the StereoPHAN central axis was aligned to the in‐room lasers, so that the dose cloud was positioned on the central axis of the detector. However, for multi‐target plans, where target dose clouds are often located away from isocenter, the phantom was shifted for QA measurements to align the target dose clouds with the detector central axis. This corresponding shift was done in the Elements software by moving the beams isocenter position on the phantom. Equation 1 was used to determine the new measurement isocenter position ($I_{m})$, from the detector central axis $\left(I_{d}\right)$, target position $\left(I_{t}\right)$, and treatment isocenter $\left(I_{p}\right)$. All positions were 3 × 1 matrices (x, y, z). The measurement and detector positions were DICOM coordinates on the phantom CT. The target and plan isocenter were DICOM coordinates on the patient planning CT.

(1) $$I_{m}=I_{d}‐R_{y}\left(\theta^{\prime }\right)\left(I_{t}‐I_{p},\;\text{where}\;\theta^{\prime }=\theta_{p}‐\theta_{m}\right)$$

The rotation matrix was included for measurements that were performed at different couch angle from planned. For example, some measurements were done at couch angle zero to minimize angular dependencies of the detectors. The 3 × 3 matrix is an elemental rotation matrix about the y‐direction (anterior‐posterior) where $\theta^{\prime }$ is the difference between the planned $\left(\theta_{p}\right)$ and measurement $\left(\theta_{m}\right)$ couch angles.

To apply shifts during measurements, the phantom was first setup to the lasers and calculated shifts ($R_{y}\left(\theta^{\prime }\right)\left(I_{t}‐I_{p}\right)$) were manually typed into the Elekta iGuide software. The iGuide software used the translational movements on the 6 Degree of Freedom Hexapod couch to apply the shifts. For shifts outside of the Hexapod range of motion, the iGuide software prompts the user to manually shift the 3‐dimensional Elekta Precise table to get close to the intended position, before automatically performing final adjustments with Hexapod couch.

In addition to validating dose on MBMS patient plans, an End‐to‐End (E2E) test was performed using the StereoPHAN phantom. The E2E test incorporated all clinical steps from initial imaging, fusion, contouring, planning, data export to record and verify system, quality assurance of the treatment plan, positioning using image guidance, and treatment delivery. The MR target insert, consisting of three spherical cavities (two 10 mm diameter and one 20 mm diameter) filed with mineral oil, was imaged using MRI and CT scanners. The images were co‐registered in MBMS and targets were contoured on the MR image set and compared with the CT data set to validate geometric accuracy. A 5‐table angle 6 FFF MBMS plan was created that delivered 18 Gy in one fraction to each target. The plan was exported to Mosaiq for delivery and pre‐treatment plan QA was performed. The in‐room imaging system was used to align the StereoPHAN with the Gafchromic film insert. The treatment plan was delivered to a Gafchromic film plane that intersects all the targets. Point dose measurements were taken with microDiamond at the center of each target. Gafchromic film and microDiamond measurements were compared to dose calculated by MBMS plan.

Following the commissioning of MBMS version 1.5, an updated version of the software 2.0 was released. A new optimizer was tested for clinical use with the following major changes: a) MLC margins can vary between −3 mm to 3 mm and change between arcs (previously a universal 1mm margin was used), b) jaws can partially cover an MLC leaf (previously fixed to the leaf edge), c) optimizer cost function focused on dose falloff as well as conformity (previously only conformity). The commissioning plans were re‐optimized with the new software and a student t‐test was used to compare plan quality metrics for the two optimizations. In addition, a subset of the plans were delivered to an SRSMapcheck and microDiamond in a StereoPHAN on a Versa HD to verify dose.

## 3 Results

### 3.1 Beam model – scatter factors

**Fig. 1:**
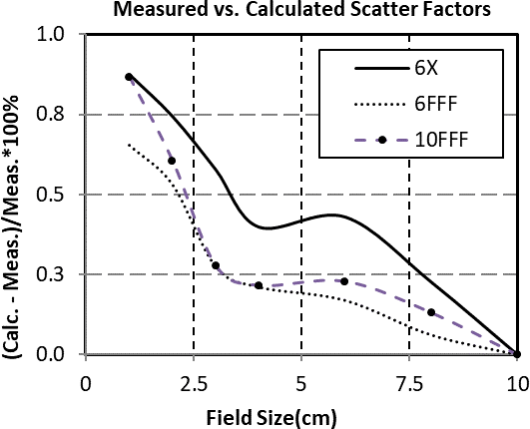
Comparison between commissioning and calculated scatter factors at 100 cm source‐to‐phantom distance and 10 cm depth. All scatter factors were normalized to unity for a 10 cm × 10 cm field.

Comparisons between measured and calculated scatter factors measured at 100 cm source‐to‐phantom distance and 10 cm depth were performed (Fig. 1). Square fields were within 1% of Elements calculated values. The absolute measured scatter factors are shown in Table 1 for comparison. The uncertainty in the measurements were calculated as two times the standard deviation of repeated measurements on separate days. It is important to note that scatter factors measurements were required without any leaves open behind the jaws. However, 1cm field size calculations could only be performed with an additional two leaves open behind each jaw that matched the width of the open leaves defining the field. Colloquially known as guard leaves, these will increase the dose for a 1 cm field by approximately 4%. This discrepancy likely contributed to the small (<10 mm) single‐target modeling error that is discussed in the dose validation section.

**Table 1:** Measured scatter factors at 100 cm SSD and 10 cm depth. Field size is nominal setting on the VersaHD. Bracketed numbers show the 2*sigma uncertainty in the last digit.

Field Size (cm)	6 X	6 FFF	10 FFF
1.0	0.677 (3)	0.696 (3)	0.680 (4)
2.0	0.806 (2)	0.830 (3)	0.843 (4)
3.0	0.847 (3)	0.872 (2)	0.894 (3)
4.0	0.879 (1)	0.901 (4)	0.923 (1)
6.0	0.928 (2)	0.945 (2)	0.958 (2)
8.0	0.970 (2)	0.977 (2)	0.982 (4)
10.0	1.000 (0)	1.000 (0)	1.000 (0)

### 3.2 Beam model – PDDs & profiles

**Fig. 2:**
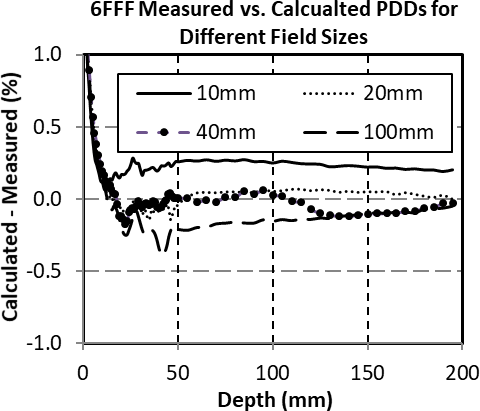
Comparison between commissioning and calculated 6 FFF percent depth doses (PDDs) at 100 cm source‐to‐phantom distance for selected field sizes.

**Fig. 3:**
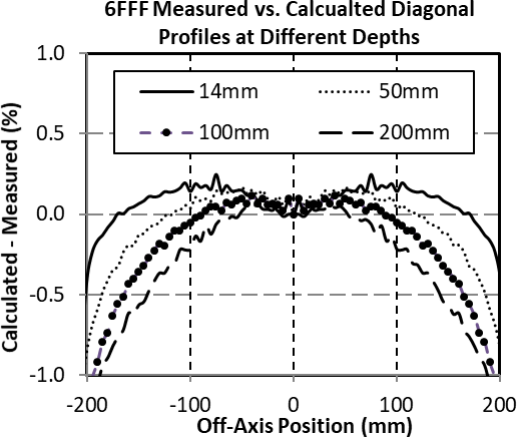
Comparison between commissioning and calculated 6 FFF diagonal profiles at 100 cm source‐to‐phantom distance for select depths.

The difference between the measured and calculated PDDS and profiles for select 6 FFF square fields and depths were compared (Fig. 2 and Fig. 3). The PDDs and Profiles were normalized to dmax and the central axis respectively. A PTW‐60019 microDiamond chamber was used for PDD scanning. Large field profiles, used in beam model generation, were scanned with the Sun Nuclear 0.125 cc chamber. Subsequent profiles down to 1 cm field size were scanned with both the microDiamond and EDGE detector for beam model validation. Measured PDDs were within 0.5% of calculated past dmax for all energies. Measured Profiles for all energies were within 0.5% of calculated for typical cranial off‐axis treatment distances (<10 cm).

### 3.3 Beam model – collimator penumbra

**Fig. 4:**
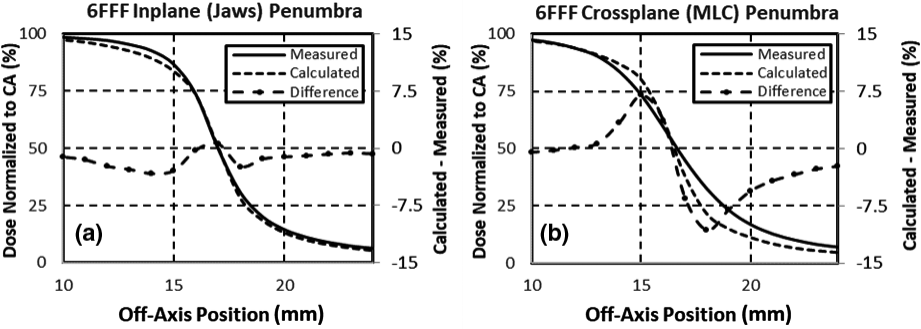
Difference between the measured and calculated 6 FFF collimator penumbras at 100 cm source‐to‐phantom distance, 10 cm depth for a 3 cm × 3 cm field size. The discrepancy is smaller in the jaw direction (a) compared with the MLC direction (b).

The measured in‐plane (jaw) penumbra was larger than the cross‐plane (MLC) penumbra, which is similar to previous publications.^11^ The difference between the measured and calculated penumbras is shown in Fig. 4. All profiles were normalized to the central axis for comparison. The differences in the jaw direction were typically < 2.0%, while the differences in the MLC direction were < 10.0%. The penumbra differences were similar for different field sizes and depths. A single penumbra model is used for both the MLC and Jaws, which resulted in a larger difference in the MLC direction.

### 3.4 Beam model – MLC DLS and transmission

The measured MLC transmissions and dynamic leaf shifts are shown in Table 2. The 10 FFF transmission was slightly lower than 6 X and 6 FFF, however, this is offset by the larger dynamic leaf shift causing increased transmission near the field edges. The transmission with the jaws closed (jaws and MLC combined) was zero percent for all energies.

**Table 2:** Measured multi‐leaf collimator (MLC) transmission and dynamic leaf shifts.

	6 X	6 FFF	10 FFF
Transmission	0.4%	0.4%	0.3%
Dynamic leaf shift	0.15	0.22	0.26

### 3.5 MBMS plan quality

**Fig. 5:**
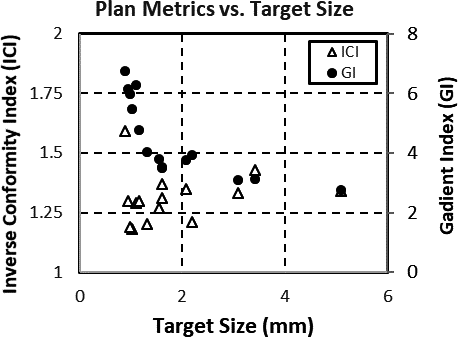
Dose metrics for single‐isocenter multi‐target plans of different target sizes created with MBMS.

The MBMS plans had GIs smaller than 5.0 and ICIs smaller than 1.5 for target diameters larger than 10 mm. For targets smaller than 10 mm, the GI increased above 5.0 for SIMT plans as shown in Figure 5. This increase occurs at approximately two times the multi‐leaf collimator (MLC) width of the Versa HD (5 mm at isocenter) and may be different for other MLCs. Due to the small size of the targets, an increase in GI corresponding to a larger 50% prescription isodose volume, has a smaller effect on V_12 Gy_ compared with a larger targets. Therefore, a higher GI is often deemed clinically acceptable, when treating small targets which may add a small volume of V_12 Gy_ with additional larger targets. Also when treated in isolation small relative amounts of V_12 Gy_ may be clinically irrelevant depending in terms of risk of radionecrosis and focal neurological deficit.^12^, ^13^ ICIs tended to be worse for irregularly shaped targets, which matches previous work showing SIMT deliveries having better normal tissue sparing for spherically shaped targets.^14^

### 3.6 MBMS dose validation

**Fig. 6:**
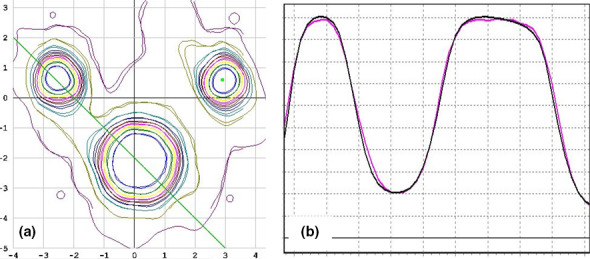
Axial film results of the end‐to‐end test. (a) Isosdose overlay between Elements‐predicted and film‐measured dose. (b) Line profile between two targets (green line in (a)).

Measured MBMS microDiamond doses had a mean difference of 0.31% compared to MBMS calculated dose with a maximum difference of 2.84%. Average per‐field pass rates measured with the SRSMapcheck in the StereoPHAN were 98.0% with a minimum of 95.5% using a 2%/1 mm/10% threshold. E2E testing showed similar results, with a microDiamond measurements within 1.5% of planned, and Gafchromic film pass rates of 98.6% and 99.6% using a 10% threshold and 3%/1 mm and 5%/1 mm gamma criteria respectively. Figure 6 shows the Gafchromic film results in the axial plane for the 3 target E2E plan. Most of the remaining failing points with the 5%/1 mm criterion were due to the pin‐holes in the Gafchromic film, which were used for registration.

When a plan was generated for only a single target, the microDiamond measured dose began to increase > 3% for targets below 10mm, possibly due to larger effect of guard leaves in smaller targets. However, this dose discrepancy was not observed when the smaller target was included in a plan with other targets. Attempts were made to manually adjust beam model scatter factors to better model single target dose, however, it was found that improvements in single‐target dose modeling would lead to larger errors in multi‐target plans. Due to the multi‐target purpose of MBMS, the decision was made to prioritize multi‐target dose modeling over single‐target.

### 3.7 MBMS version 2.0

**Fig. 7:**
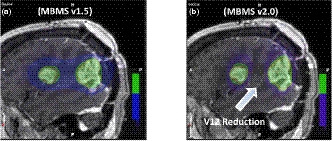
Plan comparisons between: (a) MBMS v1.5 and (b) MBMS v2.0. The updated algorithm removes the 12 Gy dose‐bridging.

The new optimization algorithm in MBMS Version 2.0 reduced ICIs by 0.05+/−0.10 [*P* < 0.01] and GIs by 0.40+/−0.65 [*P* < 0.01], with no significant changes to the PTVmin [*P* > 0.10]. Whole brain V_12 Gy_ was reduced by an average of 2.39 cc [*P* < 0.01]. The resulting reduction in whole brain V_12 Gy_ is visualized in Fig. 7 where the new optimizer eliminates the 12 Gy dose‐bridging between the two targets. A similar improvement in plan metrics in MBMS v2.0 has been observed in previous publications.^15^ Average SRSMapcheck pass rates were 98.7% [97.0% –99.8%] using relative gamma analysis (2%/1 mm/10% threshold). Measured microDiamond dose was within 1.40% of calculated for all targets.

## 4 Discussion

Due to the small field sizes used in cranial SIMT deliveries, small misalignments of the water tank may lead to sharper falloff in PDDs and lower scatter factors. For PDD measurements, the tank should be aligned to the beam axis. This will require that the water tank deviates from true level as gantry sag on the VersaHD causes the beam axis to point slightly towards the gun direction. The alignment of the beam‐axis can be verified by scanning profiles at multiple depths and ensuring the central axis of the profiles remains unchanged. Furthermore, the beam profiles should be scanned immediately prior to small field scatter factor measurements to position the detector at the local maxima within the field.

IAEA TRS‐483 provides an exhaustive list of small field correction factors for various chambers.^16^ These can be used to correct small field scatter factor measurements, particularly when comparing results between detectors. If correction factors are used for commissioning measurements, they should also be used for validation measurements. Published correction factors are typically reported for reference conditions on the central axis, while MBMS validation measurements will likely be off‐axis. The correction factor for a given field size under reference conditions may differ from validation measurement, due to changes in small field phenomenon (source blocking, volume averaging, angular dependence, etc.). Therefore, it is recommended to validate small field dosimetric accuracy with multiple detectors.

In this work, small field correction factors were not applied to the measurements. Based on TRS‐483, the microDiamond will over‐respond by approximately 1.5% for a 1.0 cm field size. Additional publications have suggested this over‐response may be up to 3.4% for a 6FFF beam on the VersaHD with a 1.0 cm field size.^17^ This over‐response will lead to an increase in the measured small field scatter factors and thereby a reduction in the delivered dose. This matches the E2E film results which were found to be within 1.5% lower than predicted for the 1.0cm target (Fig. 6). Given the expected clinical prescriptions for the small targets, this lower dose was deemed to still be ablative to the target, while the clinical organs‐at‐risk dose would be within tolerance.

Treatment planning system validation of water tank measurements was important for discovering fundamental limitations of calculation model. Specifically in Elements, a discrepancy was found in how the Versa HD MLC penumbra is modeled. It was found that error in the MLC model could lead to reduced pass rates for single field measurements at couch zero with a small collimator rotation. In these instances, the modeling error could have coherent summation leading to failing measurements along the edges of the targets. However, when the cumulative target dose from all fields in the MBMS plan was measured with couch rotations applied, the MLC modeling error had little effect on the overall dose distribution.

One of the unique challenges of SIMT commissioning was positioning the detector at the center of an off‐axis target. This was accomplished by manually applying shifts with the Hexapod couch, which has been shown to have sub‐millimeter accuracy.^18^ An initial hurdle to this technique was determining how the Hexapod coordinate system related to real‐word and TPS coordinates. It is recommended that coordinate correlations be determined ahead of QA measurements by either: a) an investigation of documentation and system settings, or b) guess‐and‐check method where shifts are applied and the resulting real‐world shifts are recorded. During commissioning the guess‐and‐check method was used to determine the coordinate relationships between: a) treatment planning system, b) six degree of freedom couch, c) in‐room imaging systems, d) QA analysis software.

As targets get farther from the central axis, the arclength error produced by a rotational error will increase sinusoidally. For small angles, the linear error will equal the radius times the angle in radians. AAPM Task Group 142 recommends a 1° collimator tolerance.^19^ A 1° collimator error would create a 0.87 mm linear error for a target 5cm away from the central axis. To reduce this error, a stricter 0.5° collimator tolerance was adopted, which was found to be consistently achievable on monthly QA. Furthermore, TG‐142 requires a 0.5° couch tolerance for SRS/SBRT, however, the clinical display only shows integers. This was overcome by enabling the “PSS” page in service mode, which reports angles in 0.1° increments. Studies show that target coverage degrades substantially when rotational errors approach 2°.^20^ Therefore, minimization of both mechanical and patient setup errors is critical, of which this can be partially accomplished by real time image guidance at each couch angle and positioning the patient with a six degree‐of‐freedom robotic couch.^21^

When creating beam and prescription protocols, it was best to create one prescription and beam protocol and fully test the planning and delivery. Once fully tested, the protocols could then be copied and modified as needed. This would help prevent unnecessary time fixing issues that may propagate through all the protocols if they are generated prior to testing.

## 5 Conclusion

The commissioning of the MBMS TPS system introduces unique challenges for physicists due in part to the small fields, off‐axis non‐coplanar beam arrangements, and high‐dose hypofractionated prescriptions. Advanced knowledge of these challenges along with the expected limitations of the MBMS beam models can add familiarity to the commissioning process. Added familiarity will hopefully lead to faster and more consistent MBMS commissioning across institutions.
